# Single Fluoroscopy Versus Transesophageal Echocardiogram: A Comparative Evaluation for Left Atrial Appendage Occlusion With LACbes Device

**DOI:** 10.1155/cdr/9933780

**Published:** 2025-10-12

**Authors:** Mingfei Li, Dawei Lin, Lei Zhang, Jianing Fan, Wenzhi Pan, Xiaochun Zhang, Daxin Zhou, Junbo Ge

**Affiliations:** ^1^Department of Cardiology, Zhongshan Hospital, Fudan University, Shanghai Institute of Cardiovascular Diseases, Shanghai, China; ^2^State Key Laboratory of Cardiovascular Diseases, Zhongshan Hospital, Fudan University, Shanghai, China; ^3^NHC Key Laboratory of Ischemic Heart Diseases, Shanghai, China; ^4^National Clinical Research Center for Interventional Medicine, Shanghai, China

**Keywords:** atrial fibrillation, fluoroscopy, left atrial appendage occlusion, transesophageal echocardiography

## Abstract

**Objectives:**

This study was aimed at evaluating the effectiveness of fluoroscopy as an intraprocedural guidance method during left atrial appendage occlusion (LAAO) using the LACbes device.

**Methods:**

The study included a cohort of 477 patients with nonvalvular atrial fibrillation (AF) who underwent LAAO. The cohort was divided into two groups: a retrospective group of 240 patients who underwent standard procedures involving both transesophageal echocardiogram (TEE) and fluoroscopy and a prospective group of 237 patients who only underwent fluoroscopy. The retrospective cohort was analyzed for device positioning, anchoring, compression, and peridevice leak (PDL) using both TEE and fluoroscopy to provide detailed insights for potential application in the prospective cohort. Clinical outcomes were compared between the two groups.

**Results:**

There were no significant differences in left atrial appendage (LAA) characteristics and other echocardiographic parameters between the two groups. Although the prospective group had a higher prevalence of implantation failure and more deployment attempts per procedure, these differences did not reach statistical significance. Notable differences were observed in fluoroscopy and operation times. Specifically, the prospective group had longer fluoroscopy times and more deployment attempts per procedure but shorter overall operation times compared to the retrospective group. Additionally, patients in the prospective group had shorter hospital stays and lower hospitalization costs. Periprocedural and 2-year follow-up complications were comparable between both cohorts, with no statistically significant differences observed in pericardial effusion, peripheral vascular complications, survival, cardiac death, PDL, or device thrombus.

**Conclusions:**

The use of fluoroscopy as the sole guidance modality for LAAO with the LACbes device demonstrated promising results in terms of efficacy and safety. It emerges as a viable and cost-effective alternative, offering a more efficient approach for LAAO.

## 1. Introduction

In recent years, left atrial appendage occlusion (LAAO) has emerged as a viable alternative to oral anticoagulation (OAC) for stroke prevention in individuals with nonvalvular atrial fibrillation (AF). It is now recommended for AF patients who are at high risk of stroke and those with contraindications to long-term anticoagulant therapy (with a classification of IIb and level of evidence C) [1]. LAAO procedures are typically performed under general anesthesia (GA), utilizing x-ray fluoroscopy and transesophageal echocardiogram (TEE) for guidance. While this approach ensures precise positioning and successful occlusion, it also comes with several drawbacks. GA increases the risks for patients with comorbidities, elevates costs, and prolongs hospital stays. However, recent evidence suggests that by using first-generation devices like the Amplatzer Cardiac Plug and second-generation devices like the Amulet, procedural success rates are notable when performed solely under fluoroscopy guidance, eliminating the need for GA [2–5]. This approach has demonstrated tolerable periprocedural complications, offering a more patient-friendly and cost-effective option.

**Figure 1:**
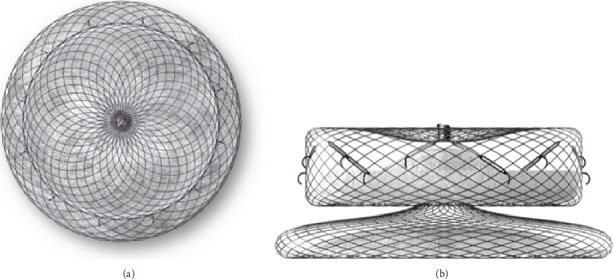
(a) The front view of LACbes occluder. (b) The side view of LACbes occluder.

Significant advancements have been made in the field of LAA occluders in recent years, with novel devices emerging in the market [6]. One such device, the LACbes (manufactured by Push Medical Equipment Co. Ltd., Shanghai, China) (Figure 1), is a recently introduced dual-seal LAA occluder that features a disc-lobe configuration [7]. Unlike the single occlusive WATCHMAN device, the LACbes device consists of two layers of barriers: a distal anchoring lobe and a proximal sealing disc, which may potentially reduce the risk of device-related complications. Compared to the initial dual-seal occluder, the Amplatzer Cardiac Plug (produced by St. Jude Medical in the United States), the LACbes device incorporates isogenous barbs positioned circumferentially within the device lobe for additional safety. These barbs are uniquely designed to minimize procedure-related complications. The safety and feasibility of the LACbes device have been established in clinical trials. The study conducted by Zhang et al., which compared the clinical outcomes of the WATCHMAN with those of the LACbes, exhibited comparable safety and efficacy of stroke prevention for AF [8]. However, whether it can be effectively utilized solely under fluoroscopic guidance remains unexplored. This question is of significant interest and warrants further investigation in the field of interventional cardiology.

In this study, we performed LAAO using the LACbes device, utilizing TEE guidance either in combination with fluoroscopy or solely relying on fluoroscopy. We further assessed the incidence of complications and subsequent outcomes during the follow-up period to evaluate the efficacy and safety of the LACbes device.

## 2. Methods

The study was conducted on a consecutive series of 477 patients referred to Zhongshan Hospital, Fudan University, for LAAO using the LACbes device. All patients had nonvalvular AF and were at high risk of stroke, with a CHA₂DS₂-VASc score of 2 or higher, and had relative or absolute contraindications for long-term OAC therapy. The study included two cohorts: a retrospective cohort of 240 patients who underwent LAAO under GA using a combination of TEE and fluoroscopy and a prospective cohort of 237 patients who underwent the procedure solely guided by fluoroscopy. Informed consent was obtained from all participants, and the study was conducted in accordance with the principles of the Declaration of Helsinki and approved by the Institutional Review Board of Zhongshan Hospital, Fudan University, Shanghai, China (2023-120R2).

### 2.1. Preprocedural Evaluation

Before the LAAO procedure, all patients in both cohorts underwent a routine TEE examination to rule out the presence of thrombi in the LAA and to assess the shape and size of the LAA. The procedure was performed via a transfemoral approach, with the preferred transseptal puncture site located in the posterior–inferior region. Intravenous heparin was administered to maintain an activated clotting time (ACT) above 250 s, and normal saline was continuously infused to ensure a left atrial pressure above 12 mmHg.

### 2.2. Retrospective Cohort

In the retrospective cohort, the procedure was performed under GA with endotracheal intubation. The TEE probe was inserted through the oral cavity, and optimal visualization of the LAA was achieved by adjusting the TEE at various angles (0°, 45°, 90°, and 135°). Measurements of the LAA's inner diameter, landing zone, and depth were taken, including both the maximum and minimum diameters. The appropriate size of the occluder device was determined by multiplying the maximum inner diameter by 120% of the landing zone measurement. After implantation, both fluoroscopy and TEE were used to assess for any peridevice leak (PDL) or pericardial effusion.

### 2.3. Prospective Cohort

**Figure 2:**
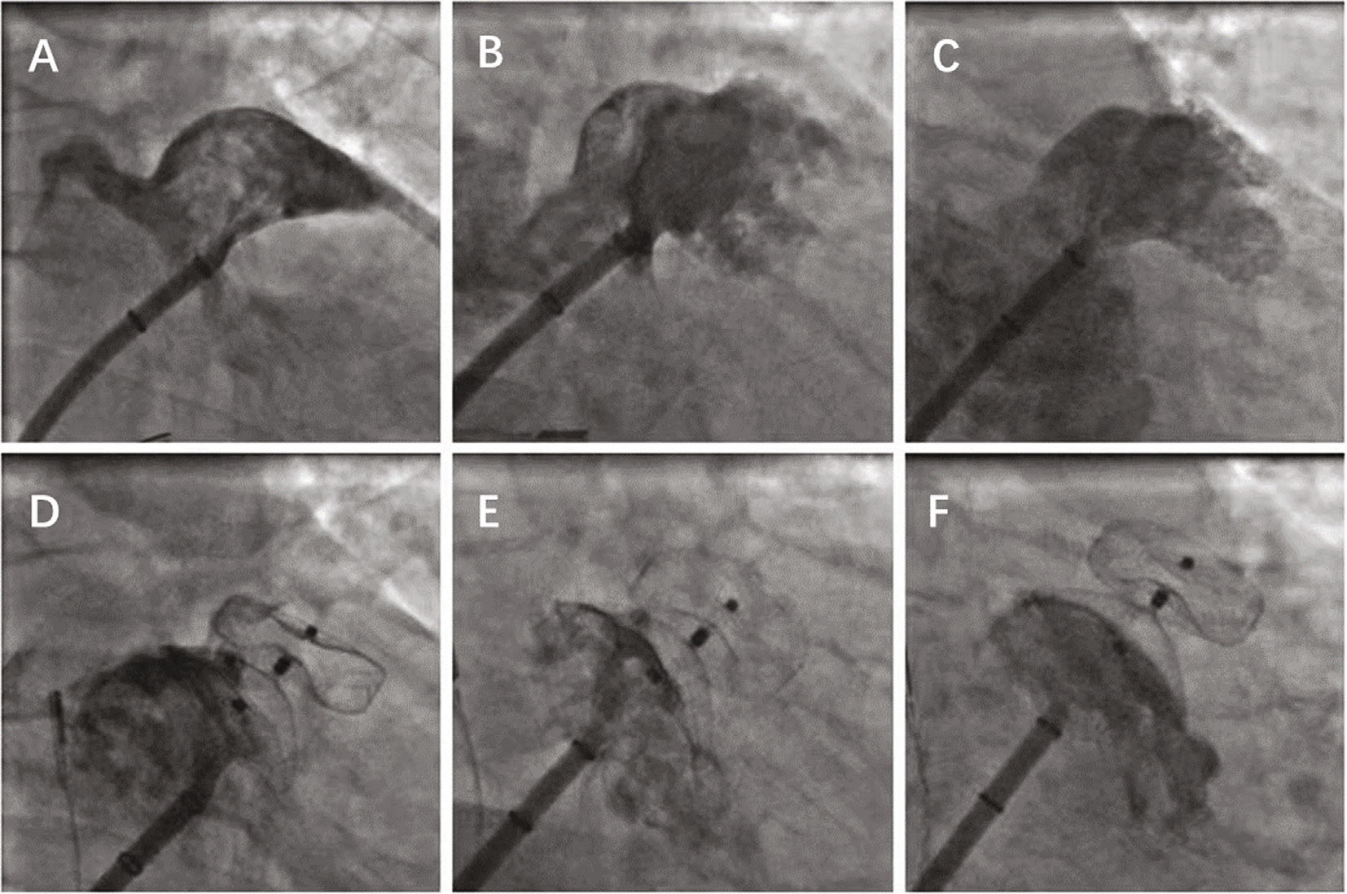
(A–C) LAA morphology was shown: (A) cranial position RAO30° + CRA20°–30°; tangential position; (B) work position RAO30° + CAU 20°–30°; (C) tangential position AP + CAU10°–20°. (D, E) None of the shunt was observed: (D) cranial position RAO30° + CRA20°–30°; (E) work position RAO30° + CAU 20°–30°; (F) tangential position AP + CAU10°–20°.

In the prospective cohort, LAAO was performed under local anesthesia using fluoroscopy and transthoracic echocardiogram (TTE) guidance. The BRK-1 transseptal needle from St. Jude Medical, along with an SL1 sheath, was carefully maneuvered under fluoroscopic guidance. The desired transseptal puncture site was confirmed using four-chamber and short-axis views on TTE. After ruling out pericardial effusion via TTE, the delivery sheath was exchanged and advanced into the left atrium. The decision to upsize the device by 4–6 mm was based on the largest diameter of the LAA ostium, measured during intraprocedural angiography at specific angles (RAO 30° + CAU 20°–30°/CRA 20°–30° and AP + CAU 10°–20°). This measurement, as shown in Figures 2, 2, and 2, was crucial for selecting the appropriate device size. The device was deployed under fluoroscopic guidance, and its positioning and functionality were evaluated before complete release, ensuring compliance with the PAST criteria (proper position, absolute anchor, separate seal, typical tire) for the LAA occlusion device, as illustrated in Figures 2, 2, and 2. This comprehensive process ensured a safe and effective procedure for LAAO in the prospective cohort.

### 2.4. Clinical Study Endpoints

The successful closure of the LAA was defined as the absence of any residual shunt or the presence of a minor residual shunt, with a shunt width not exceeding 3 mm, as assessed by TEE. Primary and secondary adverse events were meticulously documented, including death, all-cause mortality, systemic embolism, and ischemic stroke. Additionally, complications such as pericardial effusion, pericardial tamponade, hemorrhage, access-site complications, and device-related issues were recorded.

The severity of PDL was categorized as follows:

• No PDL: no detectable leak around the device.
• Mild to moderate PDL: a leak with a width of 1 mm or more but less than or equal to 3 mm.
• Severe PDL: a leak with a width of greater than 3 mm [9].

These criteria provide a comprehensive framework for evaluating the efficacy and safety of the LAA closure procedures.

### 2.5. Postprocedural Management and Follow-Up

After LAAC, patients were monitored overnight and discharged the following day if no major procedural complications occurred, such as significant pericardial effusion, tamponade, or major bleeding. In both groups, anticoagulation was recommended for 2 months, followed by dual antiplatelet therapy for up to 6 months. All enrolled patients underwent clinical follow-up via both clinical visits and telephone visits. Clinical visits were conducted using TEE to detect PDL, DRT, and successful sealing of the LAA at 3 months postoperatively. Telephone visits were conducted to find out the occurrence of endpoints every 3 months until 2 years.

### 2.6. Statistical Analysis

Continuous data were expressed as the mean ± standard deviation and were analyzed using either the Student *t*-test or the Mann–Whitney *U* test, as appropriate. Categorical data were described in terms of frequencies and percentages, and comparisons between groups were made using chi-square tests or Fisher's exact tests, as applicable. To assess differences in survival outcomes between the two groups, Kaplan–Meier survival analysis was employed. All *p* values were two-sided, and a significance level of *p* < 0.05 was considered statistically significant. The statistical analyses were performed using SPSS Version 22.0 (IBM Corporation).

## 3. Results

### 3.1. Baseline Clinical Characteristics

**Figure 3:**
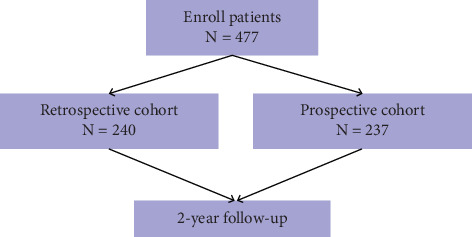
Flowchart of the cohorts.

As shown in Figure 3, a total of 477 patients who underwent the implantation of the LACbes device were included, with 237 individuals in the retrospective cohort and 240 individuals in the prospective cohort. The average age of the patients was 69.1 ± 7.9 years, with 298 males (62.5%) and 363 individuals (76.1%) with permanent AF. All patients underwent a median 2-year follow-up period. The mean CHA₂DS₂-VASc score was 3.7 ± 1.2, indicating a high risk of stroke, while the mean HAS-BLED score was 3.1 ± 1.0, suggesting a moderate risk of bleeding. The detailed clinical characteristics of the patients are presented in Table 1. As indicated in Table 2, no significant differences were observed in LAA characteristics or other echocardiogram parameters between the two cohorts. This suggests that the cohorts were well matched in terms of baseline clinical and echocardiographic features, allowing for a robust comparison of procedural outcomes and safety profiles.

### 3.2. Comparison of Intervention Information Between the Two Cohorts

As shown in Table 3, the implantation success rate in the retrospective cohort was 99.2%. This rate was marginally higher than that observed in the prospective cohort, where the success rate was 98.7%. Among the three unsuccessful cases in the prospective cohort, two cases had excessively large LAA orifices (measuring 41 and 42 mm, respectively), and one case exhibited a shallow LAA with insufficient landing zone for anchorage. However, no statistically significant difference was detected between the two cohorts in terms of procedural success (*p* > 0.05).

Regarding the incidence of implantation failure and the number of deployment attempts per procedure, a slightly higher frequency was noted in the prospective cohort. Nevertheless, this difference did not achieve statistical significance (*p* > 0.05). Notably, the fluoroscopy time was significantly longer in the prospective cohort, which also had more deployment attempts per procedure (11.3 ± 1.7 min on average, compared to 9.3 ± 2.0 min in the retrospective cohort, *p* < 0.001). In contrast, the overall operation time was shorter in the prospective cohort (45.6 ± 7.8 min on average) than in the retrospective cohort (55.5 ± 7.2 min, *p* < 0.001). Moreover, patients in the prospective cohort had a shorter hospital stay (3.1 ± 0.5 days on average, compared to 4.5 ± 0.7 days in the retrospective cohort, *p* < 0.001) and incurred significantly lower hospitalization costs ($11,825.2 ± $768.2 on average, compared to $13,189.5 ± $915.3 in the retrospective cohort, *p* < 0.001).

### 3.3. Periprocedural Complications

As shown in Table 4, no cases of cardiac tamponade, stroke or TIA, or device embolization were observed in either cohort. Pericardial effusion occurred in one patient (0.4%) in each group, with a *p* value of 0.99. Peripheral vascular complications were observed in two patients (0.8%) in each cohort, with a *p* value of 1.00. These results indicate no statistically significant differences in complication rates between the two cohorts.

### 3.4. Comparison of Adverse Events Between the Two Cohorts Post-LAAC Procedures and During the 2-Year Follow-Up

**Figure 4:**
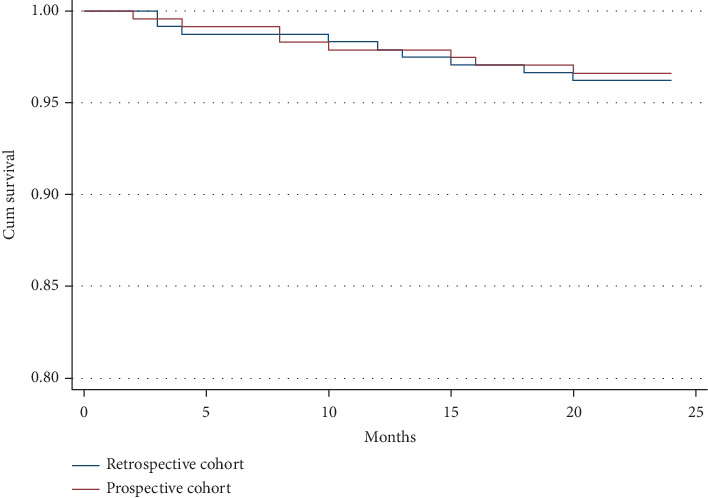
Kaplan–Meier survival graphic. There is no difference in cumulative survival between the retrospective cohort and the prospective cohort (*p* = 0.83).

The complications encountered during hospitalization and throughout a median 2-year follow-up period in the standard imaging-guided retrospective cohort versus our fluoroscopy-based prospective cohort are presented in Table 5. During the 2-year follow-up, 9 patients in the retrospective cohort and 8 patients in the prospective cohort were reported to have passed away. The Kaplan–Meier survival analysis for both groups is shown in Figure 4, revealing no significant difference in survival rates (*p* = 0.83) (Figure 4). Furthermore, there were no reports of cardiac death or device displacement in either cohort. The overall occurrence of pericardial effusion and PDL did not vary significantly between the two cohorts (*p* = 0.99 and *p* = 0.87, respectively), while the rates of mild PDL were slightly higher in the fluoroscopy cohort (8.4% vs. 6.3%). Device thrombus was reported to be equal in both the retrospective and prospective cohorts (0.8% vs. 0.8%, *p* = 1.00).

## 4. Discussion

In our comparative study, we evaluated the use of TEE and fluoroscopy for monitoring device positioning and performance during LAAO procedures. Our findings revealed that fluoroscopy demonstrated comparable effectiveness in assessing device position and stability and detecting any residual leakage. These results were subsequently utilized in a prospective study where the LAAO procedure was exclusively guided by fluoroscopy. The clinical outcomes reported in the prospective cohort during the perioperative period and follow-up further supported the reliability and safety of using fluoroscopy alone during LAAO in our institution's patient treatments. Additionally, we observed a significant reduction in operation time, hospital stay, and overall hospitalization costs in the prospective cohort compared to the retrospective cohort. This underscores the benefits of utilizing fluoroscopy as a primary monitoring tool in LAAO procedures.

To minimize radiation exposure for medical staff and reduce the potential harm to kidney function and other organs caused by contrast media in patients, LAAO procedures guided solely by TEE or intracardiac echocardiography (ICE) have become standard practice in many medical centers. These approaches have shown successful outcomes. However, they require GA, which can lead to complications such as pulmonary atelectasis, postoperative nausea, and vomiting. Additionally, GA can prolong operation times and increase the duration of hospital stays. The use of TEE also carries the risk of causing throat injuries in patients. Moreover, LAAO guided by TEE is associated with significantly higher hospitalization costs. A study by Zhang et al. explored the exclusive use of fluoroscopic guidance with the WATCHMAN device and achieved comparable results. This approach offers a potential alternative to reduce the aforementioned risks and costs associated with LAAO procedures [10].

The LACbes device is a newly introduced dual-seal LAA occluder featuring a disc-lobe design that allows for repeated retrieval and redeployment [9]. In contrast, the WATCHMAN device, a single-seal option, has been widely used and is supported by extensive evidence from clinical trials and real-world registries [11–13]. The study of Zhou et al. found no significant differences in DRT or PDL incidence between LACbes and WATCHMAN, although the incomplete device endothelialization rate in the absence of PDL was higher in the LACbes group than in the WATCHMAN group [14].

The deployment strategy for the LACbes device involves a sequential process of unsheathing and forward pushing, followed by unsheathing the anchor cylinder to create a spherical shape. This procedure requires the use of an atraumatic tip, necessitating a relatively shallow insertion of the device into the LAA. This approach is expected to reduce the risk of LAA perforation, especially when performed using a modified procedure with mild sedation. Several studies have explored the use of the LACbes device during LAAO under TEE guidance, affirming its safety and feasibility [15–16]. However, the performance of the LACbes device in LAAO procedures guided by fluoroscopy alone remains to be fully evaluated. Given the limited data available on the LACbes device, further research is warranted to fully understand its safety profile and effectiveness in clinical practice.

In our preliminary prospective study, we investigated the utility of fluoroscopy-guided left LAAO using the LACbes device. The results were promising, with a procedural success rate of 98.7%, comparable to that achieved under TEE guidance. Furthermore, there was no statistically significant difference in the occurrence of adverse events between the fluoroscopy-guided group and the TEE-guided group, both during the perioperative period and throughout the 2-year follow-up.

To ensure complete occlusion of the LAAs with the LACbes device and to identify any PDL, angiography was performed during the operation using a strategic approach. Angiography was conducted at three specific angles: cranial position RAO30° + CRA20°–30°, tangential position AP + CAU10°–20°, and working position RAO30° + CAU20°–30°. In addition to the traditional cranial and working position angles, our clinical experience has shown that the tangential position AP + CAU10°–20° could effectively visualize the opening of the atrial appendage. This angle was particularly useful for identifying any significant PDL. Our findings have been well validated within the prospective group.

This angiography strategy, employed in our center, may contribute to achieving better outcomes when utilizing fluoroscopy for LAAO procedures only. Therefore, for centers with experienced operators and a high volume of LAAO procedures, this minimalist approach could be considered for patients who have significant contraindications to GA and/or TEE.

### 4.1. Study Limitations

It is important to acknowledge several limitations in our study. Firstly, this research was conducted at a single medical center, which may have resulted in a limited sample size of patients and consequently lower clinical event rates. Additionally, while the study documented adverse events such as PDL and pericardial effusion, further analysis of prognostic factors is warranted to provide a deeper understanding of these outcomes. Furthermore, the successful execution of LAAO under fluoroscopy guidance requires the expertise of experienced operators. This suggests that less experienced teams may face challenges when attempting to replicate the achieved results.

## 5. Conclusions

The use of fluoroscopy as the sole guiding method for LAAO with the LACbes device has shown promising results in terms of efficacy and safety. This approach may offer a viable alternative, characterized by shorter operation times, reduced hospital stays, and lower hospitalization costs.

## Figures and Tables

**Table 1:** Baseline characteristics of the study population.

**Characteristics**	**Retrospective cohort (** **n** = 240**)**	**Prospective cohort (** **n** = 237**)**	**p** ** value**
Age (years)	68.4 ± 7.2	69.4 ± 8.4	0.15
Sex (male) (%)	152 (63.3)	146 (61.6)	0.70
Permanent AF (%)	182 (75.8)	181 (76.4)	0.89
Hypertension (%)	149 (62.1)	155 (65.4)	0.45
Diabetes (%)	48 (20.0)	42 (17.7)	0.52
Previous myocardial infarction (%)	42 (17.5)	40 (16.9)	0.86
Smoke (%)	46 (19.2)	33 (13.9)	0.12
Stroke (%)	98 (40.8)	98 (41.4)	0.91
Bleeding (%)	28 (11.7)	23 (9.7)	0.49
LVEF (%)	61.2 ± 5.5	61.8 ± 6.9	0.28
CHA2DS2-VASC score	3.6 ± 1.2	3.7 ± 1.2	0.44
HAS-BLED score	3.05 ± 1.1	3.1 ± 1.0	0.33

Abbreviation: LVEF, left ventricular ejection fraction.

**Table 2:** LAA characteristics in the two cohorts.

**Characteristics**	**Retrospective cohort (** **n** = 240**)**	**Prospective cohort (** **n** = 237**)**	**p** ** value**
Number of lobulations			0.88
1	227 (94.6%)	227 (95.8%)	
2	10 (4.2%)	8 (3.4%)	
3	3 (1.3%)	2 (0.8%)	
LAA opening diameter (mm)	28.45 ± 6.14	28.83 ± 6.53	0.51
LAA anchor zone diameter (mm)	23.45 ± 3.55	23.77 ± 3.79	0.35
LAA ejection rate (m/s)	0.30 ± 0.15	0.31 ± 0.16	0.44
Mitral regurgitation			0.074
0	84 (35.0%)	104 (43.9%)	
1	95 (39.6%)	74 (31.2%)	
2	45 (18.8%)	50 (21.1%)	
3	16 (6.7%)	9 (3.8%)	
LA diameter (mm)	48.44 ± 6.55	49.15 ± 7.01	0.26

Abbreviations: LA, left atrium; LAA, left atrial appendage.

**Table 3:** Comparison of intervention information between the two cohorts.

**Characteristics**	**Retrospective cohort (** **n** = 240**)**	**Prospective cohort (** **n** = 237**)**	**p** ** value**
Implantation success	238 (99.2%)	234 (98.7%)	0.64
Device resizing	2 (0.8%)	2 (0.8%)	0.99
Deployment attempts per procedure	1.5 ± 0.5	1.6 ± 0.5	0.037
Fluoroscopy time (min)	9.3 ± 2.0	11.3 ± 1.7	< 0.001
Operation time (min)	55.5 ± 7.2	45.6 ± 7.8	< 0.001
Hospital stay (days)	4.5 ± 0.7	3.1 ± 0.5	< 0.001
Hospitalized cost ($)	13,189.5 ± 915.3	11,825.2 ± 768.2	< 0.001

**Table 4:** Comparison of periprocedural complications between the retrospective cohort and the prospective cohort.

**Characteristics**	**Retrospective cohort (** **n** = 240**)**	**Prospective cohort (** **n** = 237**)**	**p** ** value**
Death			
Cardiac tamponade	0	0	NA
Pericardial effusion	1 (0.4%)	1 (0.4%)	0.99
Stroke or TIA	0	0	NA
Device embolization	0	0	NA
Peripheral vascular complication	2 (0.8%)	2 (0.8%)	1.00

Abbreviation: TIA, transient ischemic attack.

**Table 5:** Comparison of clinical outcomes and follow-up device problems between the retrospective cohort and the prospective cohort.

**Characteristics**	**Retrospective cohort (** **n** = 240**)**	**Prospective cohort (** **n** = 237**)**	**p** ** value**
Death	9 (3.8%)	8 (3.4%)	0.83
Cardiac death	0	0	NA
Pericardial tamponade	0	0	NA
Device dislocation	0	0	NA
Malignant arrhythmia	0	0	NA
Stroke	0	0	NA
Pericardial effusion	1 (0.4%)	1 (0.4%)	0.99
Residual shunt			
0 ≤ PDL ≤ 3, mm	15 (6.3%)	20 (8.4%)	0.87
PDL > 3, mm	0 (0.0%)	0 (0.0%)	
Device thrombus	2 (0.8%)	2 (0.8%)	1.00
Access-related complications	0	0	NA

Abbreviation: PDL, peridevice leak.

## Data Availability

The data that support the findings of this study are available from the corresponding authors upon reasonable request.
